# Electronic cigarette related topics with conflicting arguments in Chinese newspapers

**DOI:** 10.18332/tid/145929

**Published:** 2022-03-04

**Authors:** Joanne Chen Lyu, Peiyi Huang, Pamela M. Ling

**Affiliations:** 1 Center for Tobacco Control Research and Education, University of California San Francisco, San Francisco, United States; 2 Department of Psychology, School of Social Sciences, University of Mannheim, Mannheim, Germany

**Keywords:** e-cigarettes, topics with conflicting arguments, Chinese newspapers

## Abstract

**INTRODUCTION:**

While debates on e-cigarettes are mainly conducted in scientific fora, media are the most accessible information source for the public, shaping their perceptions of health issues. This study is the first to examine e-cigarette related topics with conflicting arguments presented in Chinese newspapers.

**METHODS:**

The Chinese terms for ‘e-cigarettes’ were searched in a widely used Chinese news database *Wisenews*. Content analysis of the full text of 639 news articles was conducted to identify topics with conflicting arguments and examine whether the dominant argument in each topic changed across four time periods from 2004 to 2019.

**RESULTS:**

Twelve e-cigarette related topics with conflicting arguments were identified. The most frequently reported topic was health impact of e-cigarettes, followed by impact of secondhand aerosol exposure, smoking cessation, relative health impact of e-cigarettes compared to cigarettes, and e-cigarette policies outside China. At the same time, the price was the least frequently reported topic. Overall, negative arguments outnumbered positive arguments in the study period. The dominant arguments within many topics changed across time periods; however, within the topics of relative health impact of e-cigarettes compared to cigarettes, taste/flavor, and economic prospects of the industry, positive arguments were more frequently reported in almost all periods. Within the topics of addiction, policies in China, and policies outside China, negative arguments were more frequently reported in virtually all periods.

**CONCLUSIONS:**

Though overall the dominant argument about e-cigarettes and health was ‘e-cigarettes are harmful’, in the early time periods, e-cigarettes were reported as ‘harmless’ or even ‘healthy’. As China began to regulate e-cigarettes, the reporting on e-cigarettes more frequently included the ‘e-cigarettes are harmful’ argument. The consistent, more frequent reporting of ‘good e-cigarette taste/flavor’ has the potential to attract young people to e-cigarette products. The increased reporting on policies unfavorable to e-cigarettes aligned with the growing number of regulations restricting e-cigarettes.

## INTRODUCTION

Electronic cigarettes (e-cigarettes), known by many different names such as ‘e-cigs’, ‘vapes’, and electronic nicotine delivery systems (ENDS) etc., are devices that produce an aerosol by heating a liquid that usually contains nicotine, flavorings, and chemicals^1^. First appearing in the Chinese market in 2003, e-cigarettes have gained increasing popularity worldwide. The value of the global e-cigarette market reached $11.5 billion in 2018 and is anticipated to increase significantly in the next few years^2^. Meanwhile, e-cigarettes are the subject of debate in the public health field. Studies on health effects or health risk of e-cigarettes have been inconsistent and sometimes come to contradictory conclusions^3,4^. For instance, while some studies found that e-cigarette use may worsen asthma, bronchitis, and cough, including among non-smoking young people^5,6^, other studies found that after switching to e-cigarettes, smokers with asthma or chronic obstructive lung disease reported improved symptoms^7,8^.

While the British Royal College of Physicians^9^ and National Academies of Sciences, Engineering, and Medicine^10^ concluded that vaping is likely far less harmful than smoking cigarettes, the American College of Physicians called for more research into the short- and long-term health consequences of e-cigarette use.^11^ Another topic with conflicting opinions related to e-cigarettes is whether or not to recommend e-cigarettes for tobacco cessation in adults. Though a growing body of evidence indicates that e-cigarettes can help with smoking cessation, others note that the evidence is still not definitive^12-14^. As of 2021, almost all systematic reviews or meta-analyses have concluded that more carefully designed and scientifically sound studies are needed on e-cigarettes and their health consequences^11,15^. In the absence of scientific consensus, e-cigarettes will continue to be the subject of debate.

Medical and academic debates conducted in scientific fora are generally inaccessible to the public. The general public most frequently accesses this kind of information through the media, which shapes public awareness, perceptions of and opinions about issues^16^. Previous research found that exposure to e-cigarette news shapes harm perceptions of e-cigarettes and intentions to use the product^17^. Another study assessing effects of exposure to conflicting news headlines about e-cigarettes on US adults found that those exposed to negative e-cigarette news headlines reported increased beliefs about e-cigarette harms and decreased ideas about the benefits of e-cigarette use compared with those who were exposed to positive headlines^18^. Therefore, the media coverage of e-cigarettes, especially the conflicting information about e-cigarettes, deserves examination. China has special status in the e-cigarette industry: China manufactures 95% of the world’s e-cigarettes^19^, and investors have predicted it will be ‘eventually the biggest market of e-cigarettes’^20^. However, communication about e-cigarettes in China is understudied. To the best of our knowledge, no studies have addressed the conflicting information about e-cigarettes presented in Chinese media.

This study will fill the gap by identifying e-cigarette-related topics with conflicting arguments and the dominant argument for each topic presented in Chinese newspapers from 2004–2019 (i.e. from the appearance of the first e-cigarette news report in China to when the study began). China promulgated its first nationwide e-cigarette policy in 2018 and has strengthened regulation of e-cigarettes since then. Therefore, findings of this study will also provide a context for the ongoing e-cigarette policy making.

## METHODS

### Dividing 2004–2019 into four time periods

Government policies significantly influence Chinese media’s coverage of relevant issues^21^. Through a thorough review of e-cigarette and cigarette policies in China in the past two decades, three most significant policies in the period of 2004–2019 were identified^22^ : the first regulation about e-cigarettes in China in 2007^23^; the first nationwide smoking ban in public places in 2011^24^; and the first national-level e-cigarette regulation in 2018^25^. We divided the 16 years between 2004–2019 into four periods marked by these policies: 2004–2006, 2007–2010, 2011–2017 , and 2018–2019.

### Data and coding method

Chinese translations of ‘e-cigarettes’ were searched as keywords in a widely used Chinese news database *Wisenews* ^26,27^ from 6 March 2004 when the first e-cigarette news article appeared in China to 31 July 2019 when the study began. *Wisenews* is a full-text news database providing access to more than 600 newspapers, magazines, and websites from China, Hong Kong, Macao, and Taiwan as well as some regional newspapers from the US, and it is widely accepted as a valid database to examine Chinese media coverage. The search limited results to mainland Chinese newspapers only. Newspapers are an appropriate medium to examine e-cigarette reporting due to the important status of newspapers in the media landscape in China. Compared to newspapers, social media and other commercial news websites have far fewer opportunities to generate original reporting, and more often replay or amplify newspaper news stories on their platforms^28^. Thus, newspapers are more likely to set the agenda for other media formats in China^28-30^. We removed 547 news articles that mentioned e-cigarettes only in passing and 80 news articles that were irrelevant to e-cigarettes or duplicates. 639 news articles from 124 newspapers, containing both market-oriented newspapers and Party newspapers, were included in the analyses. Market-oriented newspapers, which are generally more liberal and critical than Party newspapers^31^, accounted for most of the newspaper e-cigarette reports from 2004–2019. Among the 639 analyzed news articles, 552 news articles (86.4%) were from 105 market-oriented newspapers, and 87 news articles (13.6%) were from Party newspapers.

We used the combination of deductive and inductive approaches to develop the codebook. Based on a literature review of the topics and themes of e-cigarette reporting in China^32^, Korea^33^, the US^34^, and the UK and Scotland^35^, we developed a list of themes. Some of the themes centered around one topic but included conflicting opinions, such as ‘e-cigarettes are a healthier alternative to cigarettes’^35^ versus ‘e-cigarettes are not harmful or less harmful than conventional cigarettes^33^. Another example of conflicting opinions was ‘e-cigarette vaping does not affect others’ versus ‘e-cigarette vaping affects others’^33^. In this analysis, we defined these contrasting themes as ‘arguments’. Each study topic included two conflicting arguments. For example, the topic of addiction has two arguments: ‘e-cigarettes are not addictive’, and ‘e-cigarettes are addictive’. Topics that did not involve differing opinions (such as e-cigarettes’ impact on youth, which was universally reported as a negative) were removed from the list for this analysis. A randomly selected sample of 100 news articles (15% of the sample) were read to refine the list of topics by adding new arguments and deleting arguments that were not identified in the e-cigarette articles from Chinese newspapers. Another 10% of the news articles were pilot coded to test the exhaustiveness and applicability of the codebook. Through iterative rereading, discussion and revision, we identified 12 topics that included 24 conflicting arguments in total. The topics, arguments, and examples of the arguments are elaborated in Table 1. Content analysis was conducted on the full text of each article. The unit of analysis was a news article. Once an argument was identified within a news article, no matter how many times it was mentioned, it was coded as 1; otherwise, it was coded as 0. When a news article mentioned more than one argument, each of the mentioned arguments was coded as 1. Two trained graduate students coded all the articles for the presence of the arguments. A random subsample of 200 articles (31%) was double-coded to check the inter-coder reliability (Krippendorf’s alpha), ranging from 0.80 to 0.96 for each of the twenty-four arguments. Any disagreements were resolved through thorough discussion before coding the remaining articles. Disagreements that could not be resolved by discussion between the two coders were discussed with a senior researcher who participated in the development of the codebook until consensus was achieved or a final decision about the coding was made. The data we analyzed in this study are publicly accessible newspaper articles and did not involve human subjects. According to the policy of the Institutional Review Board (IRB) of our institution, this study did not need IRB approval.

**Table 1:** Topics and arguments on electronic cigarettes in Chinese newspapers 2004–2019

*Topics*	*Examples*
**Health impact of e-cigarettes**	
**Not harmful** E-cigarettes have some short-term or long-term health benefits. E-cigarettes are harmless for health.	*E-cigarettes allow smokers to enjoy the fun of smoking under the premise of being healthy.* (Beijing Science and Technology News, 10 November 2004)
**Harmful** E-cigarettes harm health. E-cigarettes are tobacco. E-cigarettes increase health risks.	*The World Health Organization's newly released Global Tobacco Epidemic Report 2019 states: ‘There is no doubt that e-cigarettes are harmful and should be regulated.’* (Information Times, 31 July 2019)
**Relative health impact compared to cigarettes**	
**Less harmful than cigarettes** E-cigarettes are presented as healthier, less harmful than combustible cigarettes/other inhaled substances/other tobacco products. E-cigarettes do not contain the harmful substances in cigarettes.	*Although the amount of nicotine contained in each of our e-cigarettes is higher than that of regular cigarettes, the amount broken down into each puff is lower than that of regular cigarettes.* (Xi´an Evening News, 23 November 2006)
**As harmful or more harmful than cigarettes** E-cigarettes do not have advantages in term of health compared to tobacco products. E-cigarettes are harmful as tobacco.	*Some brands of e-cigarettes release carcinogenic elements. Compared to cigarettes, they do more harm to the body.* (Wuhan Evening News, 8 May 2019)
**Price**	
**Not more expensive than cigarettes** E-cigarettes help save money than buying cigarette products. E-cigarettes are not more expensive or even cheaper compared to combustible cigarettes. Low cost of buying e-cigarettes or a starter pack.	*Benefit 3: Huge savings, only the price of one pack of cigarette, it can be used for most of the year.* (Jiangnan Metropolitan News, 25 December 2010)
**More expensive than cigarettes** E-cigarettes are more expensive compared to combustible cigarettes.	*This so-called e-cigarette vape is actually more expensive than smoking traditional cigarettes: A variety of fresh equipment itself is not cheap. In addition, the price of those different liquid smoke flavors is also far more expensive than a pack of traditional cigarettes.* (Yangcheng Evening News - National Edition, 3 March 2018)
**Addiction**	
**Not addictive** E-cigarettes are not addictive. The ingredients in e-cigarettes are not additive.	*Benefit 1: No harm to the body, no addiction, you can smoke when you want.* (Jiangnan Metropolitan News, 25 December 2010)
**Addictive** E-cigarettes are addictive. E-cigarettes contain additive ingredients.	*E-cigarette use by children, adolescents and young adults has been proven to be unsafe because it is highly addictive and can impair brain development.* (Global Times, 26 July 2019)
**Use safety**	
**Safe** E-cigarettes are safe or safer than tobacco cigarettes. E-cigarettes have no lighting fire or smoke.	*[E-cigarettes] do not produce ‘secondhand smoke’ or pollute the public environment or burn no fire hazards. It is safe and scientific.* (People's Daily Market Edition, 3 June 2005)
**Dangerous** E-cigarette vaping is dangerous. E-cigarettes have safety risks. E-cigarettes can explode.	*E-cigarettes also have the risk of explosion, high temperature burns, etc.* (Beijing Business Today, 29 July 2019)
**Impact of secondhand aerosol exposure**	
**No adverse effects on others** E-cigarettes do not expose others to secondhand smoke. E-cigarettes have no ashes. Others have no secondhand smoking concerns. E-cigarettes are cleaner and more environmentally friendly than cigarettes.	*[E-cigarette] does not produce ‘secondhand smoke’, does not pollute the public environment, has no fire. It is a healthy alternative to cigarettes that has far-reaching implications for public health and environmental protection.* (China Trade News, 13 December 2015)
**Has adverse effects on others** E-cigarette vaping causes others’ concerns and affects one’s social acceptance by others. Secondhand smoke of e-cigarettes contains toxic and hazardous substances.	*Secondhand smoke from e-cigarettes, which are more harmful than traditional cigarettes, is a new source of air pollution.* (The Beijing News, 18 March 2019)
**Taste/flavor**	
**As good or even better than cigarettes** E-cigarettes have more palatable flavors than cigarettes. The taste of e-cigarettes is better and more refreshing than cigarettes.	*It has the same appearance as cigarettes, similar taste to cigarettes, and even more than the general taste of cigarettes.* (Western Business News, 14 March 2014)
**Not as good as cigarettes** Cigarettes taste better than e-cigarettes.	*But the first time Mr. Yu used this ‘alternative’, he felt particularly astringent. He even felt a little nauseous.* (Shanghai Evening Post, 31 May 2010)
**Smoking cessation**	
**Are effective tools to quit smoking** E-cigarettes are effective cessation devices and can help quit or reduce cigarette/tobacco smoking.	*E-cigarette sales often claim that e-cigarettes help smokers quit smoking.* (Modern Health Post, 15 October 2008)
**Not effective tools to quit smoking** E-cigarettes fail to help quit smoking. Lack of scientific evidence that show the effectiveness of e-cigarettes as smoking cessation aids.	*In fact, to WHO's knowledge, there are no rigorous, peer-reviewed studies showing that e-cigarettes are a safe and effective nicotine replacement therapy.* (Modern Health Post, 15 October 2008)
**Policies in China**	
**Are favorable to e-cigarettes** The policies in Mainland China do not clearly regulate or even promote e-cigarette manufacture, sales, marketing, or use.	*In recent years, China's tobacco control efforts are also strengthening, not only from May 1 this year in public places, a complete ban on smoking, but also increased support for the development of tobacco cessation and control products, some high-tech smoking cessation and control products to give policy support and guidance.* (China Commercial Times, 2 March 2013)
**Are unfavorable to e-cigarettes** Mainland China restricts e-cigarette manufacture, sales, marketing, or use.	*The State Tobacco Department reacted quickly and issued a notice on August 28 this year in conjunction with the State Administration of Market Supervision and Administration to prohibit the sale of e-cigarettes to minors and protect them from abuse.* (Information Times, 7 December 2018)
**Policies outside China**	
**Are favorable to e-cigarettes** The policies in other countries or regions (outside Mainland China) do not clearly regulate or even promote e-cigarette manufacture, sales, marketing, or use.	*[In UK] These devices do not contain tobacco and therefore are not subject to the regulatory requirements for tobacco products. In addition, because e-cigarettes are not classified as medical devices, they cannot be regulated according to the medical device regulations.* (Wei Hui Bao, Shanghai, 23 March 2014)
**Are unfavorable to e-cigarettes** Other countries or regions (outside Mainland China) restrict e-cigarette manufacture, sales, marketing, or use.	*According to a proposal announced by US FDA, manufacturers must obtain approval from FDA before selling e-cigarettes. Manufacturers are prohibited from claiming that e-cigarettes are safer than other tobacco products, providing free trial products, or selling e-cigarettes to people under 18.* (Beijing Times, 26 April 2014)
**Regulation effectiveness**	
**Regulations are effective** Policies regulate e-cigarette manufacture, sales, marketing or use as intended or policies are effectively enforced.	*A passenger was administratively detained for using e-cigarettes on a plane, which is against the smoking regulations on airplanes.* (Beijing Morning News, 12 October 2018)
**Regulations are ineffective** Policies don’t regulate e-cigarette manufacture, sales, marketing or use as intended or policies are not effectively enforced.	*Because of the short history of e-cigarettes, governments of different countries lack effective regulation.* (Shanghai Morning Post, 28 August 2014)
**Economic prospects of the industry**	
**Positive** People speculate a positive e-cigarette economy ahead. The e-cigarette stock market goes up. Traditional tobacco and many other companies enter the e-cigarette market.	*E-cigarettes are now the most competitive product in the cigarette replacement market and are growing rapidly, and many traditional cigarette manufacturers have begun to venture into this area.* (Securities Times, 26 November 2012)
**Negative** People speculate a negative e-cigarette economy and uncertainties ahead. The e-cigarette stock market goes down.	*This is an industry with an uncertain future, and once e-cigarettes are treated as cigarettes and taxed at a high rate, their cost is bound to increase dramatically.* (Securities Times, 21 November 2013)

## RESULTS

**Figure 1:**
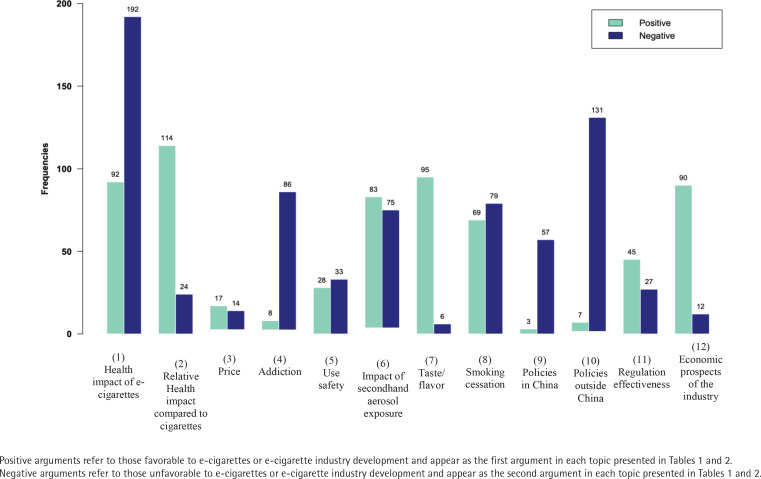
Number of positive and negative arguments within each topic in Chinese newspapers 2004–2019

Among the 12 topics with conflicting arguments (Table 2), the most frequently reported topics were health impact of e-cigarettes (n=284), impact of secondhand aerosol exposure (n=158), smoking cessation (n=148), relative health impact compared to cigarettes (n=138), and policies outside China, which was about whether policies outside China were favorable to e-cigarettes or not (n=138). Economic prospects of the industry, taste/flavor, and addiction were also hot topics, with each being reported in around 100 news articles. There were also topics with conflicting arguments presented in newspapers around use safety (n=61), policies in China (n=60), and the price (n=31), which was the least frequently reported among the 12 topics with conflicting arguments. Comparing the number of conflicting arguments within each topic in the whole study period of 2004–2019, it was observed that the arguments that were unfavorable to e-cigarettes or e-cigarette industry development (shortened as ‘negative arguments’ thereafter) were reported more frequently than arguments favorable to e-cigarettes or e-cigarette industry development (abbreviated as ‘positive arguments’ thereafter) within the topics of health impact of e-cigarettes, addiction, use safety, smoking cessation, policies in China, and policies outside China, while positive arguments were more reported than negative arguments within the topics of relative health impact compared to cigarettes, price, impact of secondhand aerosol exposure, taste/flavor, regulation effectiveness, and economic prospects of the industry. Overall, negative arguments outnumbered positive arguments in the e-cigarette related newspaper reporting on topics with conflicting opinions (Figure 1).

**Table 2:** Frequency of electronic cigarette related topics with conflicting arguments in Chinese newspapers 2004–2019

*Topics*	*2004–2006*	*2007–2010*	*2011–2017*	*2018–2019*	*Total*
**Health impact of e-cigarettes**					
Not harmful	61	12	18	1	92
Harmful	9	26	72	85	192
Total	70	38	90	86	284
**Relative health impact compared to cigarettes**					
Less harmful than cigarettes	46	13	46	9	114
As harmful or more harmful than cigarettes	2	7	9	6	24
Total	48	20	55	15	138
**Price**					
Not more expensive than cigarettes	5	3	8	1	17
More expensive than cigarettes	8	2	1	3	14
Total	13	5	9	4	31
**Addiction**					
Not addictive	1	3	3	1	8
Addictive	1	4	29	52	86
Total	2	7	32	53	94
**Use safety**					
Safe	24	0	2	2	28
Dangerous	1	0	11	21	33
Total	25	0	13	23	61
**Impact of secondhand aerosol exposure**					
No adverse effects on others	49	8	23	3	83
Has adverse effects on others	0	15	22	38	75
Total	49	23	45	41	158
**Taste/flavor**					
As good or even better than cigarettes	17	12	35	31	95
Not as good as cigarettes	1	0	5	0	6
Total	18	12	40	31	101
**Smoking cessation**					
Are effective tools to quit smoking	35	15	16	3	69
Not effective tools to quit smoking	7	8	26	38	79
Total	42	23	42	41	148
**Policies in China**					
Are favorable to e-cigarettes	0	0	3	0	3
Are unfavorable to e-cigarettes	0	12	6	39	57
Total	0	12	9	39	60
**Policies outside China**					
Are favorable to e-cigarettes	0	1	4	2	7
Are unfavorable to e-cigarettes	0	5	71	55	131
Total	0	6	75	57	138
**Regulation effectiveness**					
Regulations are effective	0	6	19	20	45
Regulations are ineffective	7	2	9	9	27
Total	7	8	28	29	72
**Economic prospects of the industry**					
Positive	2	2	67	19	90
Negative	0	2	7	3	12
Total	2	4	74	22	102

The dominant arguments (i.e. the argument reported more frequently than its conflicting argument within the same topic) of six topics changed across the four time periods: 2004–2006, 2007–2010, 2011–2017, and 2018–2019. They were health impact of e-cigarettes, price, use safety, impact of secondhand aerosol exposure, smoking cessation, and regulation effectiveness. In addition, a pattern was observed within the topics of health impact of e-cigarettes, use safety, impact of secondhand aerosol exposure, and smoking cessation: the dominant arguments were the positive arguments in the first period, 2004–2006, but changed to the negative arguments starting in the second period, 2007–2010, or the third period, 2011–2017. Take the topic of use safety for example, in the period 2004–2006, the positive argument ‘the use of e-cigarettes is safe’ was reported in 24 news articles, while only one news article mentioned the negative argument ‘the use of e-cigarettes is dangerous’. However, after the period of 2007–2010, the dominant argument about use safety became ‘the use of e-cigarettes is dangerous’. In the period 2011–2017, the ratio of positive argument to negative argument was 2:11, and in the period 2018–2019, the ratio was 2:21. Within the topic of regulation effectiveness, a different pattern was observed: the negative argument ‘the regulation of e-cigarettes is ineffective’ was the dominant argument in the first period but replaced by the corresponding positive argument after that. As to the topic of price, which was about whether e-cigarettes are more expensive than cigarettes, the two conflicting arguments alternately were the dominant argument.

Within the topics of relative health impact compared to cigarettes, taste/flavor, and economic prospects of the industry, the positive arguments that were favorable to e-cigarettes or e-cigarette industry development were the dominant arguments in almost all of the four time periods. Specifically, they were the arguments of ‘e-cigarettes are less harmful than cigarettes’, ‘taste/flavor of e-cigarettes is as good or even better than cigarettes’, and ‘economic prospects of the industry is positive’. In contrast, for the topics of addiction, policies in China, and policies outside China, the arguments that were unfavorable to e-cigarette or e-cigarette industry development were the dominant arguments in almost all of the four time periods. Specifically, they were the arguments of ‘e-cigarettes are addictive’, ‘Mainland China's current policies on e-cigarettes are unfavorable to e-cigarettes’, and ‘policies on e-cigarettes outside Mainland China are unfavorable to e-cigarettes’.

## DISCUSSION

In Chinese newspapers between 2004–2019, the most frequently reported e-cigarette related topics with conflicting arguments were around health impact, including health impact of e-cigarettes, relative health effects of e-cigarettes compared to cigarettes, impact of secondhand aerosol exposure, and efficacy of e-cigarettes as smoking cessation aids. This finding aligns with research on e-cigarette media coverage in many countries^32-35^. However, while there was a relative balance between positive arguments and negative arguments on e-cigarette efficacy of smoking cessation and impact of secondhand aerosol exposure (reflected in the number of news articles), the dominant arguments related to the health impact of e-cigarettes in the study period were ‘e-cigarettes are harmful’ and ‘e-cigarettes are less harmful than cigarettes’. In addition, we found that in the early time of the first period, 2004–2006, the original wording used to describe e-cigarettes was usually ‘healthy’ rather than ‘not harmful’, the term used more frequently later in the study period. For instance, it was reported that ‘the advent of e-cigarettes marks the arrival of the era of healthy smoking’ (Henan Legal Daily, 24 August 2006). The positive framing (rather than negative framing) of the articles referring to the ‘healthiness of e-cigarettes’ may reflect the early influence of the e-cigarette industry on media, which may have encouraged inclusion of promotional language in media content^36^. In addition, it was noted that in the period of 2007-2010, the reporting containing the arguments ‘e-cigarettes are not harmful’ and ‘e-cigarettes are less harmful than cigarettes’ was sharply reduced; in contrast, ‘e-cigarettes are harmful’ became the dominant argument about health impact of e-cigarettes, especially in the period of 2018-2019. This may be because since 2018, China has formally started restricting e-cigarettes^25,37^ and in the current media system, press content would be expected to be aligned with policies^38^. However, these data cannot address whether or not the policy agenda directly influenced e-cigarette news reporting.

We observed an increase in reporting about e-cigarette regulation and policies in China and in other countries in 2011–2019 that paralleled the emerging prominence of e-cigarette regulation in China. Interestingly, despite the absence of e-cigarette policies in China before 2018, most newspaper articles contained the argument that regulation of e-cigarettes is effective. This may be because some of the reporting on regulation effectiveness was not about China. Another possible reason may be the press self-censorship in China that restrains media from criticizing the government^39^. Meanwhile, the majority of the reports on e-cigarette policies were about policies in other countries with large amount of reporting on restriction policies, such as banning sale of e-cigarettes to people under the age of 18 years in the United Kingdom in 2014, prohibiting e-cigarettes indoors in New York in 2017, and the U.S. Food and Drug Administration policy to prohibit sales of most flavored e-cigarettes in stores in 2018. On the one hand, this highlighted the lack of e-cigarette regulation in China; on the other hand, since news coverage has impact on governmental policy elites in terms of their perception of importance placed on an issue and the need for policy action^24^, a large number of reports on policies that restrict e-cigarette sales and use in other countries may presage e-cigarette legislation in China. A series of e-cigarette regulations after 2018 seemed to address this, such as the issuance of Notice on Further Protecting Minors from Electronic Cigarettes that banned online sales of e-cigarettes in 2019^37^, and the announcement by the State Council of China on amending the regulations of the Tobacco Monopoly Law, requiring ‘e-cigarettes and other new tobacco products to be implemented with reference to the relevant regulations of cigarettes’ in 2021^40^.

Economic prospects of the e-cigarette industry were predominantly reported as positive, even when restrictions on e-cigarette sales were subsequently enacted. This may be due to the fact that the e-cigarette use rate in China was low^41^ and the huge e-cigarette manufacturing industry was mainly driven by the continuously increasing demand from markets outside China. In addition, this study found that in terms of e-cigarette taste/flavor, the articles including the argument ‘e-cigarettes taste as good or even better than cigarettes’ greatly outnumbered the articles including, ‘e-cigarettes do not taste as good as cigarettes’ both overall and in each of the four time periods. This finding complements the results of a previous study on adult e-cigarette users in China, which reported that a common reason for e-cigarette use was non-irritating or palatable taste/flavors^42^. More than half of the participants in that study also mentioned that an advantage of e-cigarettes was the wide variety of flavors. Some e-cigarette users also articulated that the similarity between tobacco flavored e-liquid and cigarettes made it easier for them to switch from combustible cigarettes to e-cigarettes^42^. At the same time, flavored e-cigarettes drove the US youth vaping epidemic with more than 82.9% of young users reporting that they used flavored e-cigarettes, according to the 2020 National Youth Tobacco Survey in the US^43^. Therefore, the finding that ‘better taste’ information dominated newspaper coverage, may raise concerns for its impact on susceptible young people. Though China has accelerated its regulation on e-cigarettes in recent years, specific regulations on e-cigarette flavors are lacking. This issue deserves more legislative discussion and should be put on the agenda of e-cigarette policy making. Last but not least, it was also noted that the price of e-cigarettes compared to that of cigarettes was least frequently reported. This may be because the price of e-cigarettes in China is usually comparable to that of combustible cigarettes^44^. It is also consistent with the finding of another study that prices of e-cigarettes were least influential on cigarette smokers’ intention to use e-cigarettes in China^45^.

### Limitations

This study focused on the e-cigarette reporting in newspapers. Though newspapers remain the primary medium for analysis of media coverage on issues due to their status as agenda setters for other media^29,30^, social media have become an important information source, especially for young people in China. To have a more thorough and comprehensive understanding of media coverage of e-cigarettes, e-cigarette-related information on social media platforms should be examined. In addition, the ways that different media platforms influence each other in e-cigarette reporting may deserve exploration.

## CONCLUSIONS

There was much conflicting information about e-cigarettes presented in Chinese newspapers in the past almost two decades: the main e-cigarette related topics with conflicting arguments focused on health impact of e-cigarettes, and the price was the least frequently reported topic. The dominant argument about ‘health impact of e-cigarettes’ changed from ‘e-cigarettes are not harmful’ in the early time to ‘e-cigarettes are harmful’ later. Though conflicting information existed, the dominant voice about e-cigarettes in all four time periods from 2004–2019 was: e-cigarettes are less harmful than cigarettes; e-cigarettes are addictive; taste/flavor of e-cigarettes is as good as or even better than cigarettes; policies on e-cigarettes both in Mainland China and in other countries are unfavorable to e-cigarettes; the economic prospects of the e-cigarette industry are positive. Despite the limitations mentioned above, this study is the first to examine e-cigarette related conflicting information presented in Chinese media, providing important empirical data and a unique perspective to understand the public’s e-cigarette information exposure, which may potentially influence public perceptions of e-cigarette use. Moreover, as the first attempt in this line of research, findings of this study also provide valuable reference for future monitoring of media representation of conflicting information around e-cigarettes to inform e-cigarette policy making.

## Data Availability

Data sharing is not applicable to this article as no new data were created.
